# Why the Physiologic Salt Solution?

**Published:** 1913-11

**Authors:** 


Why the Physiologic Salt Solution? Hugh H. Trout, last December, read a paper before the Southern Surgical and Gynæcologic Assn. advocating the use of ordinary water in rectal injections to restore blood pressure, on the ground that there is no more indication for administering a salt solution by this route than by the mouth, that there is often an indication to reduce salines in the system and that absorption would be more rapid if the injected liquid were of lower osmotic tension than the blood. He also calls attention to the importance of accuracy in the saline contents of liquids injected into the veins or subcutaneously. Hæmolysis occurs sharply if blood is placed in solutions at or below 5 per mille of NaCl or in those at or above 11 per mille. While a teaspoonful holds approximately 5 c.c. and corresponds approximately to as many grams of water, Trout has found that a teaspoonful of salt may mean anything between 115 and 270 grains (about 7½-18 grams). Various comments on Trout's advocacy of ordinary water are favorable. For several years we have carried or kept on hand for powders of salt containing the right amount to make a 6:1000 solution in a quart (5 2/3 grams to 946 c.c.) Still better, we consider stock solutions corresponding to the various saline constituents of blood plasma, minus calcium. These solutions are, for the sake of convenience, made up in ten-fold strength and are the same as may be used in carrying out the Trunecek treatment of arteriosclerosis.

